# Supplementary material for: Child defecation and feces management practices in rural Bangladesh: Associations with fecal contamination, observed hand cleanliness and child diarrhea
Source: PLoS One. 2020 Jul 20;15(7):e0236163. doi: 10.1371/journal.pone.0236163 (PMC7371197; doi:10.1371/journal.pone.0236163)
Supplement: S1 Table — (DOCX) [file pone.0236163.s001.docx]

**Table S1. Mean *E. coli* prevalence and log_10_ MPN count by season (summer vs. monsoon vs. winter season)**

|  | Caregiver hand rinse | | | Child hand rinse | | | Stored drinking water | | | Visible dirt on caregiver hands | | Visible dirt on child hands | | 2-day prevalence of diarrhea | | 7-day prevalence of diarrhea | |
| --- | --- | --- | --- | --- | --- | --- | --- | --- | --- | --- | --- | --- | --- | --- | --- | --- | --- |
|  | N | % positive (n) | Mean log_10_ MPN (SD) | N | % positive (n) | Mean log_10_ MPN (SD) | N | % positive (n) | Mean log10 MPN (SD) | N | % (n) | N | % (n) | N | % (n) | N | % (n) |
| Summer | 588 | 69 (406) | 1.18 (0.95) | 583 | 65 (380) | 1.06 (0.89) | 506 | 74 (376) | 1.26 (0.89) | 588 | 67 (396) | 588 | 78 (458) | 580 | 8.1 (47) | 580 | 12 (69) |
| Monsoon | 1041 | 76 (787) | 1.13 (0.92) | 1027 | 77 (787) | 1.21 (0.93) | 908 | 87 (794) | 1.50 (0.94) | 1041 | 66 (689) | 1041 | 78 (817) | 1026 | 6.8 (70) | 1026 | 11 (116) |
| Winter | 795 | 77 (795) | 1.15 (0.92) | 1013 | 79 (796) | 1.19 (0.89) | 905 | 77 (700) | 1.19 (0.90) | 1033 | 67 (690) | 1033 | 80 (830) | 1020 | 7.9 (82) | 1020 | 13 (129) |

MPN: Most probable number; SD: Standard deviation.
