# Supplementary material for: Child defecation and feces management practices in rural Bangladesh: Associations with fecal contamination, observed hand cleanliness and child diarrhea
Source: PLoS One. 2020 Jul 20;15(7):e0236163. doi: 10.1371/journal.pone.0236163 (PMC7371197; doi:10.1371/journal.pone.0236163)
Supplement: S2 Table — (DOCX) [file pone.0236163.s002.docx]

**Table S2: Subgroup analysis by summer vs. monsoon/wet vs. winter/dry season on *E. coli* prevalence**

|  |  | All seasons | | | Summer season ^a^ | | | Monsoon/wet season ^a^ | | | Winter/dry season ^a^ | | | Interaction  p-value ^b^ |
| --- | --- | --- | --- | --- | --- | --- | --- | --- | --- | --- | --- | --- | --- | --- |
|  |  | N | % (n ) | Adjusted PR ^c^ (95% CI) | N | % (n ) | Adjusted PR ^c^ (95% CI) | N | % (n) | Adjusted PR ^c^ (95% CI) | N | % (n) | Adjusted PR ^c^ (95% CI) |  |
| Presence of *E. coli* on caregiver hands |  |  |  |  |  |  |  |  |  |  |  |  |  |  |
|  | Unsafe child defecation | 2088 | 76 (1593) | 1.05 (0.97, 1.14) | 447 | 72 (321) | 1.10 (0.94, 1.30) | 807 | 76 (616) | 1.03 (0.93, 1.14) | 834 | 79 (656) | 1.09 (0.98, 1.21) | 0.25 |
|  | Unsafe child feces disposal | 2055 | 76 (1562) | 1.02 (0.96, 1.09) | 447 | 73 (325) | **1.17 (1.01, 1.37)** | 784 | 76 (593) | 1.01 (0.91, 1.10) | 824 | 78 (644) | 1.03 (0.94, 1.15) | **0.04** |
| Presence of *E. coli* on child hands |  |  |  |  |  |  |  |  |  |  |  |  |  |  |
|  | Unsafe child defecation | 2030 | 77 (1563) | **1.11 (1.04, 1.19)** | 445 | 68 (301) | 1.13 (0.96, 1.32) | 790 | 79 (624) | **1.08 (1.02, 1.17)** | 795 | 80 (638) | **1.10 (1.02, 1.20)** | 0.77 |
|  | Unsafe child feces disposal | 2016 | 77 (1554) | **1.11 (1.03, 1.20)** | 442 | 69 (304) | **1.22 (1.02, 1.47)** | 777 | 79 (611) | 1.07 (0.98, 1.17) | 797 | 80 (639) | 1.07 (0.97, 1.18) | 0.31 |
| Presence of *E. coli* in stored drinking water |  |  |  |  |  |  |  |  |  |  |  |  |  |  |
|  | Unsafe child defecation | 1824 | 83 (1513) | **1.10 (1.02, 1.18)** | 391 | 79 (310) | **1.28 (1.11, 1.47)** | 707 | 90 (633) | **1.09 (1.01, 1.17)** | 726 | 79 (570) | 1.08 (0.97, 1.21) | **0.03** |
|  | Unsafe child feces disposal | 1821 | 83 (1504) | **1.10 (1.03, 1.18)** | 389 | 79 (307) | **1.24 (1.06, 1.43)** | 705 | 89 (630) | **1.08 (1.01, 1.15)** | 727 | 78 (567) | 1.04 (0.95, 1.15) | **0.03** |
| Visible dirt on caregiver hands |  |  |  |  |  |  |  |  |  |  |  |  |  |  |
|  | Unsafe child defecation | 2078 | 67 (1390) | 0.93 (0.82, 1.06) | 447 | 68 (306) | 0.96 (0.78, 1.17) | 807 | 66 (536) | 0.96 (0.84, 1.10) | 834 | 67 (556) | 0.94 (0.81, 1.08) | 0.73 |
|  | Unsafe child feces disposal | 2040 | 68 (1396 ) | 1.04 (0.89, 1.21) | 447 | 70 (314) | 1.13 (0.89, 1.42) | 784 | 68 (531) | 1.02 (0.87, 1.19) | 824 | 68 (559) | 1.03 (0.88, 1.20) | 0.53 |
| Visible dirt on child hands |  |  |  |  |  |  |  |  |  |  |  |  |  |  |
|  | Unsafe child defecation | 2023 | 82 (1676) | **1.15 (1.03, 1.26)** | 445 | 83 (369) | **1.15 (1.03, 1.28)** | 790 | 83 (653) | **1.12 (1.02, 1.24)** | 795 | 83 (660) | **1.20 (1.03, 1.38)** | 0.89 |
|  | Unsafe child feces disposal | 2004 | 82 (1650) | **1.12 (1.02, 1.22)** | 442 | 83 (365) | **1.12 (1.02, 1.24)** | 777 | 82 (635) | **1.09 (1.01, 1.18)** | 797 | 82 (656) | **1.17 (1.01, 1.34)** | 0.61 |
| 2-day prevalence of diarrhea |  |  |  |  |  |  |  |  |  |  |  |  |  |  |
|  | Unsafe child defecation | 2017 | 8.7 (176) | 1.44 (0.48, 4.26) | 422 | 8.1 (36) | 1.45 (0.49, 4.32) | 786 | 8.4 (66) | 1.42 (0.52, 3.86) | 789 | 9.4 (74) | 1.55 (0.59, 4.09) | 0.40 |
|  | Unsafe child feces disposal | 1996 | 9.0 (180) | 1.11 (0.42, 2.89) | 438 | 8.2 (36) | 1.09 (0.36, 3.29) | 770 | 8.8 (68) | 0.89 (0.36, 2.09) | 788 | 9.6 (76) | 1.55 (0.57, 4.21) | 0.57 |
| 7-day prevalence of diarrhea |  |  |  |  |  |  |  |  |  |  |  |  |  |  |
|  | Unsafe child defecation | 2017 | 13 (253) | 1.17 (0.63, 2.18) | 422 | 12 (51) | 1.41 (0.59, 3.41) | 786 | 12 (96) | 1.06 (0.57, 1.96) | 789 | 14 (106) | 1.19 (0.59, 2.37) | 0.76 |
|  | Unsafe child feces disposal | 1996 | 13 (268) | 1.73 (0.88, 3.40) | 438 | 12 (53) | 1.91 (0.76, 4.80) | 770 | 13 (102) | 1.47 (0.72, 3.02) | 788 | 14 (113) | 1.88 (0.86, 4.13) | 0.94 |

PR= Prevalence ratio; CI: Confidence interval

^a^ Summer season: March to June; Monson/wet season: July to October; Winter/dry season: November to February

^b^ We developed a model that includes interaction terms between the exposure variable and the indicator variables for season and tested the significance of the interaction terms with the Wald test. A p-value <0.2 was considered evidence of interaction

^c^ Multivariable model includes all variables associated with individual outcomes (*E. coli* in samples, visible dirt on caregiver and child hands and 2-day and 7-day diarrhea prevalence) in bivariate analyses at p<0.2 level.
